# Promising hollow multi-shelled structures: discovering the temporal-spatial ordering

**DOI:** 10.1093/nsr/nwaa106

**Published:** 2020-06-03

**Authors:** Rose Amal

**Affiliations:** School of Chemical Engineering, University of New South Wales, Australia

Harvesting incident photons is the first step in a photo-induced process such as photocatalysis, photovoltaics or photodetection, which crucially governs the process performance. Unfortunately, extensive energy losses are observed in this step due to severe surface reflections and limited optical paths of the short-wavelength light [1]. Thus, it is desirable to develop an advanced nano/macro structure that could efficiently harvest light of different wavelengths and minimize excessive photo-reflection and photo-heating [2,3].

**Figure 1.**
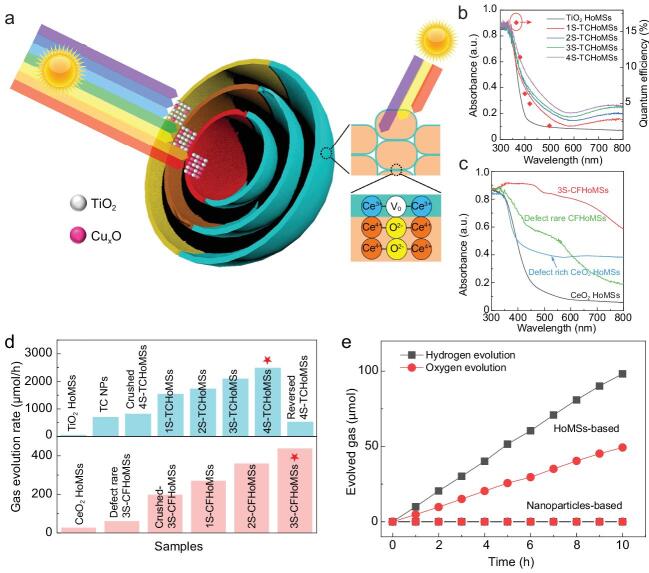
(a) Illustration of TCHoMSs and CFHoMSs for efficient sequential harvesting of solar light. UV-Vis absorption curves of (b) TCHoMSs with different shell numbers and apparent quantum efficiency (red dots) of 4S-TCHoMSs at different wavelengths and (c) 3S-CFHoMSs, CeO_2_ HoMSs and corresponding samples with surface defect control. (d) Hydrogen evolution activity and oxygen evolution activity of TCHoMSs- and CFHoMSs-related samples under 300 W Xe lamp irradiation at 281 K, and (e) overall water-splitting performance of 4S-TCHoMSs and CFHoMSs under 300 W Xe lamp irradiation at 281 K. Adapted from [5].

In a recently published article in the *National Science Review*, Dan Wang's group from the Institute of Process Engineering, Chinese Academy of Sciences, and his collaborators put forward the new concept of a sequential light-harvesting nano/macro structure that can be realized by two different types of hollow multi-shelled structures (HoMSs): the TiO_2_-Cu_x_O HoMSs (TCHoMSs) and the CeO_2_-CeFeO_3_ HoMSs (CFHoMSs). Inspired by the antenna system of cyanobacteria [4], TCHoMSs were synthesized from different band-gap materials from the outside-in. The heterogeneous shells enable the weakly penetrable short-wavelength light and the strongly penetrable long-wavelength light to be preferentially absorbed by the outer and inner HoMSs components, respectively (Fig. 1a) [5]. Results from UV-Vis spectra confirm that the overall light absorption efficiency is elevated with an increased number of shells, as the inner shells enhance visible light absorption (Fig. 1b). In addition, surface modification of 0D subunits of the 3D CFHoMSs is beneficial for harnessing the light, as the defect-rich surface is sensitive to light with longer wavelengths, promoting full-spectrum absorption (Fig. 1c). Consequently, two stages of sequential light harvesting on the micro- and nano-scale are demonstrated: shell structures with controlled compositions and subunit surfaces with tailored oxygen vacancies.

The temporal-spatial ordering of the HoMS delivers the impressive property of sequential light harvesting. The greatly enhanced photocatalytic performance of the newly designed HoMSs highlights the crucial role of sequential light harvesting in photocatalysis (Fig. 1d). The facile structural design means overall water splitting can be achieved by combining two types of HoMSs in a particle suspension system. Such a performance is not attainable by traditional nanoparticles with the same compositions (Fig. 1e). The authors also demonstrate that the shell sequence is crucial to performance. Reversing the shell order in TiO_2_-Cu_x_O HoMSs sees a decrease in photocatalytic hydrogen evolution activity and apparent quantum efficiency.

The recent discovery by Dan Wang's group reported in *Nature Reviews Chemistry* [3] is the first to demonstrate the temporal-spatial ordering concept of HoMSs. Since 2009, Wang's group has pioneered HoMS fabrication. They developed a sequential templating approach (STA) as a universal method for fabricating hollow multi-shell structures. The breakthrough in synthesis methodology was rapidly embraced by scientists worldwide, with various groups adopting the approach where HoMSs were identified as promising materials for many applications. Up until June 2018 approximately 72.5% of HoMSs were synthesized using the STA [4]. The ability to induce kinetic and thermodynamic control during the STA enables precise control over the micro-nano structure of HoMS, thus evoking successful research in the field. Reflecting on the past and looking into the future, the discovery of temporal-spatial ordering is timely and can be considered as a milestone in the hollow-structured material field, providing immense opportunity in the design of smart materials.

Overall, the temporal-spatial-ordered HoMSs are capable of sequential light harvesting, an effect that promotes enhanced photo-energy conversion efficiency compared to simple hollow materials or randomly stacked nanoparticles. The material synthesis platform provides an excellent strategy for designing photo-based materials. Additionally, the new temporal-spatial ordering concept endows unique characteristics on HoMSs, which introduces a new material route for smart cascade catalysis, responsive drug delivery, dynamic energy storage, and hybrid chemical separation.

***Conflict of interest statement*.** None declared.
